# Case report: Successful treatment with contezolid in a patient with tuberculous meningitis who was intolerant to linezolid

**DOI:** 10.3389/fmed.2023.1224179

**Published:** 2023-10-19

**Authors:** Zhe Xu, Jing Zhang, Tingting Guan, Guichuan Wan, Chao Jiang, Linchuan Lang, Lianzhi Wang

**Affiliations:** Harbin Chest Hospital, Harbin, Heilongjiang Province, China

**Keywords:** contezolid, linezolid, tuberculous meningitis, case report, antituberculosis drugs

## Abstract

Tuberculous meningitis (TBM) is the most common form of central nervous system tuberculosis (TB) and the most severe form of extrapulmonary TB. It often presents with non-specific symptoms initially and has a high mortality and disability rate. With good central nervous system penetration, linezolid is recommended for treating drug-resistant, severe, or refractory tuberculous meningitis in China. Despite the benefits of linezolid on TBM treatment, the adverse effects of long-term therapy, such as myelosuppression, peripheral neuritis, and optic neuritis, are notable and can be severe and even life-threatening, leading to discontinuation and compromising treatment expectations. Contezolid is a novel oxazolidinone antibacterial agent approved by the National Medical Products Administration of China in 2021, which has a more favorable safety profile than linezolid in terms of myelosuppression and monoamine oxidase inhibition. Here we first report a case of TBM in a patient who was intolerant to antituberculosis treatment with linezolid and achieved good efficacy and safety results after the compassionate use of contezolid. Given the widespread use of linezolid in TB treatment and the potential risks for long-term use, multi-center prospective controlled clinical trials in TB and TBM patients are needed to investigate the appropriate use of contezolid further.

## Introduction

Tuberculous meningitis (TBM) is a diffuse, nonsuppurative inflammatory disease of the soft meninges and cerebral arachnoid caused by *Mycobacterium tuberculosis* (MTB) invading the subarachnoid space, which may also invade the brain parenchyma and cerebral vessels. TBM is the most severe form of MTB infection and accounts for approximately 1% to 5% of all TB cases; TBM remains one of the most serious and deadly diseases in the world, with high mortality rates (up to 50% in adults and 20% in children) despite aggressive anti-TB treatment (1).

Linezolid is an oxazolidinone antibacterial drug with good anti-MTB activity and potent antibacterial activity against drug-resistant strains. It has good tissue penetration into the cerebrospinal fluid, its bioavailability is almost 100%, and there is no cross-resistance between linezolid and other commonly used anti-TB drugs (2). The 2019 *WHO consolidated guidelines on drug-resistant tuberculosis treatment* (3) and *Chinese expert consensus on multidrug-resistant tuberculosis and Rifampicin-resistant tuberculosis treatment* (4) classified linezolid as a Group A drug in long-course regimens, and *Consensus on linezolid in the treatment of tuberculosis (2022 update)* (2) recommended it for drug-resistant, severe and refractory TBM. However, the adverse effects of linezolid are common in antituberculosis (anti-TB) treatment, including myelosuppression (anemia, thrombocytopenia, leukopenia), peripheral neuritis, and optic neuritis, which can be severe and even life-threatening, leading to discontinuation and compromising the expectation of the treatment (5).

**Figure 1:**
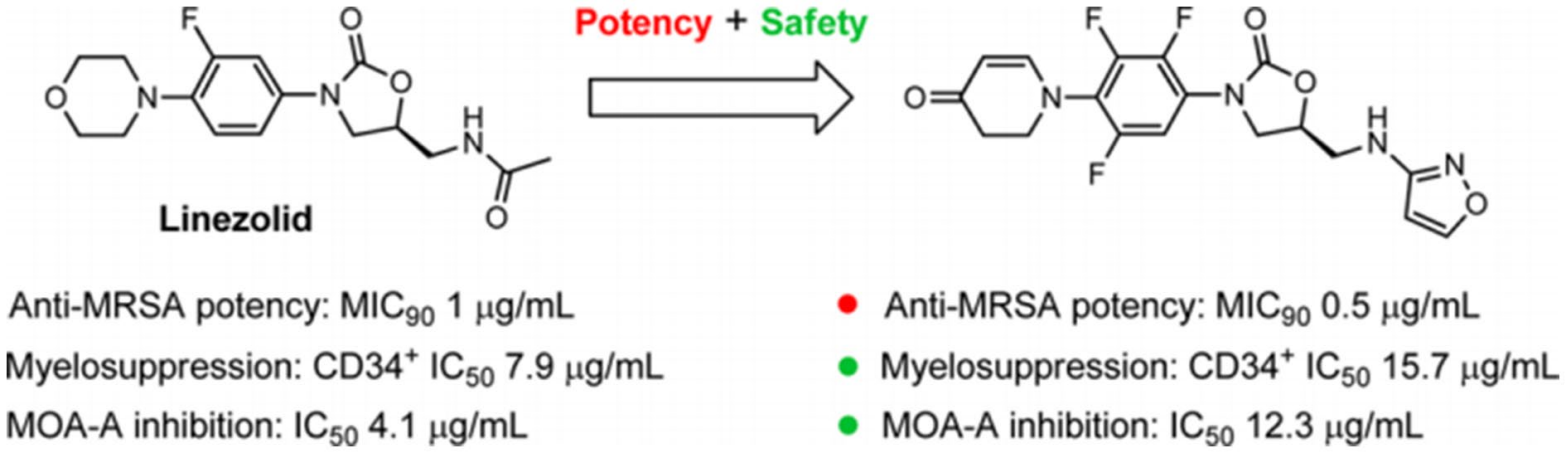
Linezolid and a novel oxazolidinone agent (S)-5-((isoxazol-3-ylamino) methyl)-3-(2,3,5-triflfluoro-4-(4-oxo-3,4-dihydropyridin-1(2H)-yl) phenyl) oxazolidin-2-one (MRX-I). Reprinted with permission from Gordeev et al. (6). Copyright (2014). American Chemical Society.

Contezolid is a novel oxazolidinone antibiotic, approved by the National Medical Products Administration of China in 2021, for treating complicated skin and soft tissue infections caused by *Staphylococcus aureus* (methicillin-sensitive and -resistant strains), *Streptococcus pyogenes* or *Streptococcus agalactiae*. Compared with linezolid, contezolid has a better safety profile and a significantly lower immunosuppressive effect and monoamine oxidase inhibition (6) (Figure 1).

In this work, we report a case of TBM who was intolerant to linezolid and then compassionately treated with contezolid for eight months to provide a reference for future anti-TB treatment and research.

## Case description

A 69-year-old female patient, 165 cm tall and weighing 43 kg, was admitted to the hospital on February 4, 2022, due to “fever and slurred speech for over one month.” On December 27, 2021, the patient developed a fever without an apparent cause, with the highest body temperature reaching 39.7°C, accompanied by slurred speech and ptosis of the left eyelid. She was referred to a general hospital due to progressive deterioration and losing consciousness. On January 12, 2022, *Mycobacterium tuberculosis* was detected by next-generation sequencing (NGS) of cerebrospinal fluid (CSF); therefore, the patient was further transferred to an infectious disease hospital. The drug resistance gene of *Mycobacterium tuberculosis* was then detected in the CSF using the real-time fluorescence PCR melting curve method, and the real-time PCR amplification testing result for *Mycobacterium tuberculosis* was positive. The testing results for drug-resistant gene mutation sites of *Mycobacterium tuberculosis*, including rifampicin, isoniazid, ethambutol, and fluoroquinolones, were all negative. Following one week of anti-TB therapy with isoniazid, rifampicin, pyrazinamide, ethambutol, and linezolid, the fever was relieved, but generalized peripheral edema and acute renal failure were diagnosed. After three cycles of hemodialysis in a kidney disease hospital, her renal function recovered. She then came to our hospital for further treatment. Since the onset of the illness, the patient has had poor appetite and sleep and denied any history of drug and food allergies when she was previously healthy.

The laboratory findings on admission to our hospital were as follows: hematologic parameters: white blood cell (WBC) count: 7.51 × 10^9^/L, red blood cell (RBC) count: 3.37 × 10^12^/L↓, and hemoglobin (HB): 102 g/L↓; biochemistry: alanine aminotransferase (ALT): 127.6 U/L↑, aspartate aminotransferase (AST): 53.6 U/L↑, total bilirubin (TBIL): 54.10 μmol/L↑, direct bilirubin (DBIL): 36.20 μmol/L↑, blood urea nitrogen (BUN): 2.81 mmol/L, creatinine (Cr): 57 μmol/L, and uric acid (UA): 142 μmol/L↓; urinalysis: no abnormality; routine CSF testing: pressure: 130 mmH_2_O, appearance: colorless and transparent, chloride (Cl): 118 mmol/L, adenosine deaminase (ADA): 4.2 U/L, glucose (Glu): 1.88 mmol/L↓, protein (Pr): 0.94 g/L↑, WBC count: 22 × 10^6^/L↑, Pandy’s reaction (+), and tryptophan reaction (+). The patient was initially diagnosed with TBM (treatment-negative) [CSF testing: smear (−), and culture (−)]; mild anemia; hepatic impairment, and jaundice.

**Figure 2:**
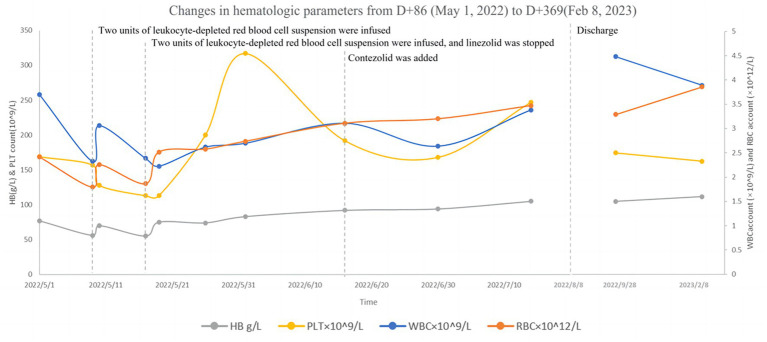
Changes in hematologic parameters from D + 86 (May 5, 2022) to D + 369 (February 8, 2023).

**Figure 3:**
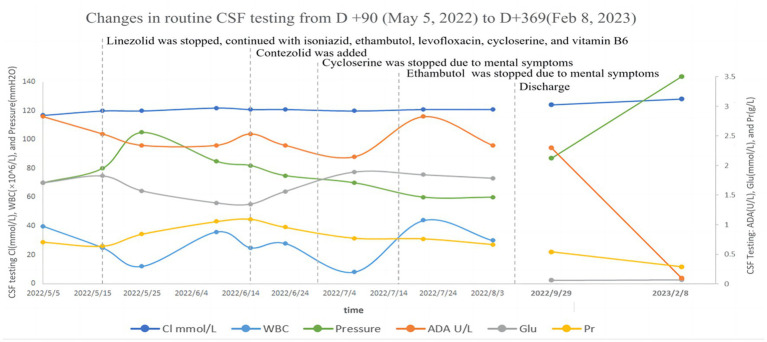
Changes in routine CSF testing from D + 90 (May 5, 2022) to D + 369 (February 8, 2023).

Following liver protection therapy, the patient was administered anti-TB treatment with isoniazid, rifampicin, ethambutol, linezolid, and levofloxacin, but she developed liver dysfunction again after rifampicin was restarted. The anti-TB therapy was finalized on D + 20 (“D + X” refers to “the X-th day after admission “): isoniazid (0.6 g, qd, po), ethambutol (0.75 g, qd, po), levofloxacin (0.6 g, qd, po), linezolid (0.6 g, qd, po), cycloserine (0.25 g in the morning and 0.5 g at night, po) and vitamin B6 (50 mg in the morning and 100 mg at night, po). Lumbar punctures were repeated every 7–10 days, and an intraspinal injection of 0.1 g of isoniazid +5 mg of dexamethasone was given. At this time, routine blood tests showed WBC 3.47 × 10^9^/L↓, RBC 2.73 × 10^12^/L↓, and HB 87 g/L↓. Hepatic and renal dysfunction did not recur, and fever and other clinical symptoms gradually resolved. After more than two months of anti-TB therapy with the above regimen, all CSF testing gradually improved, and the CSF TB bacterial culture remained negative. On D + 86, hematologic parameters showed WBC 3.69 × 10^9^/L↓, RBC 2.41 × 10^12^/L↓, and HB 77 g/L↓. The patient developed numbness and pain in both lower limbs and suffered from nausea, vomiting, and loss of appetite. On D + 94, hematologic parameters showed WBC 2.32 × 10^9^/L↓, RBC 1.79 × 10^12^/L↓, and HB 56 g/L↓. Two units of leukocyte-depleted red blood cell suspension were infused, and leucocyte-stimulating agents were taken orally. On D + 95, hematologic parameters showed WBC 3.05 × 10^9^/L↓, RBC 2.25 × 10^12^/L↓, and HB 70 g/L↓. On D + 102, hematologic parameters showed WBC 2.38 × 10^9^/L↓, RBC 1.86 × 10^12^/L↓, and HB 55 g/L↓. Two units of leukocyte-depleted red blood cell suspension were again infused, and linezolid was discontinued. Routine blood test indicators then gradually improved. On D + 132, hematologic parameters showed WBC 3.10 × 10^9^/L↓, RBC 3.10 × 10^12^/L↓, and HB 92 g/L↓ (Table S1 in Supplementary material) Nausea and vomiting disappeared, and numbness and pain in both lower limbs were improved slightly, but CSF indicators were worsened. On the same day (D + 132), contezolid (0.4 g, bid, po) was added with the patient’s consent, and other medications were continued as before. One week later, CSF indicators were improved. The CSF was then reexamined every two weeks, with continued improvement of the relevant indices. During this period, hematologic parameters continued to improve. However, as mental symptoms such as confusion and dysphoria gradually worsened, cycloserine was discontinued on D + 144, and ethambutol was discontinued on D + 161. Following these interventions, the mental symptoms were improved significantly. Changes in routine blood and CSF testing are shown in Figures 2, 3 (the detailed data were listed in Tables S1, S2 in Supplementary material).

**Figure 4:**
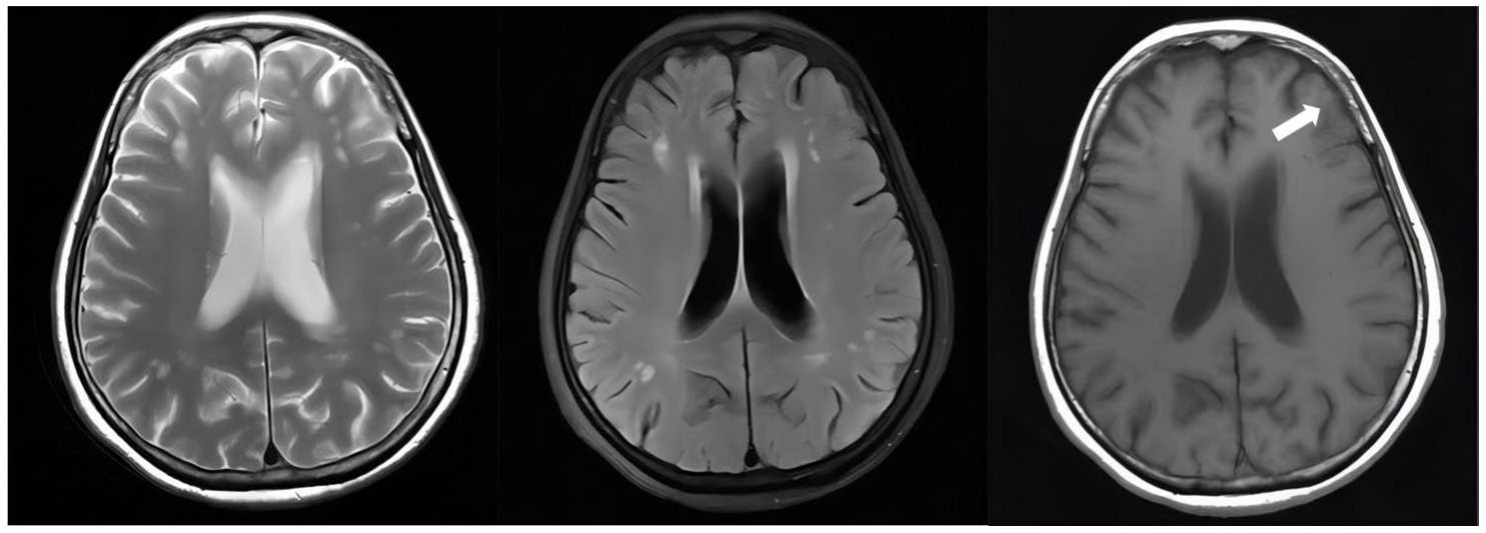
Unenhanced brain MRI scan on April 19, 2022(D + 74): Multiple infarcts and ischemic foci were seen bilaterally in the periventricular and frontoparietal white matter; there were abnormally enhancing lesions in the right ambient cistern, left sylvian cistern and adjacent brain parenchyma, which was considered a diagnosis of TBM, especially by referring to her medical history; also there was mild brain atrophy.

**Figure 5:**
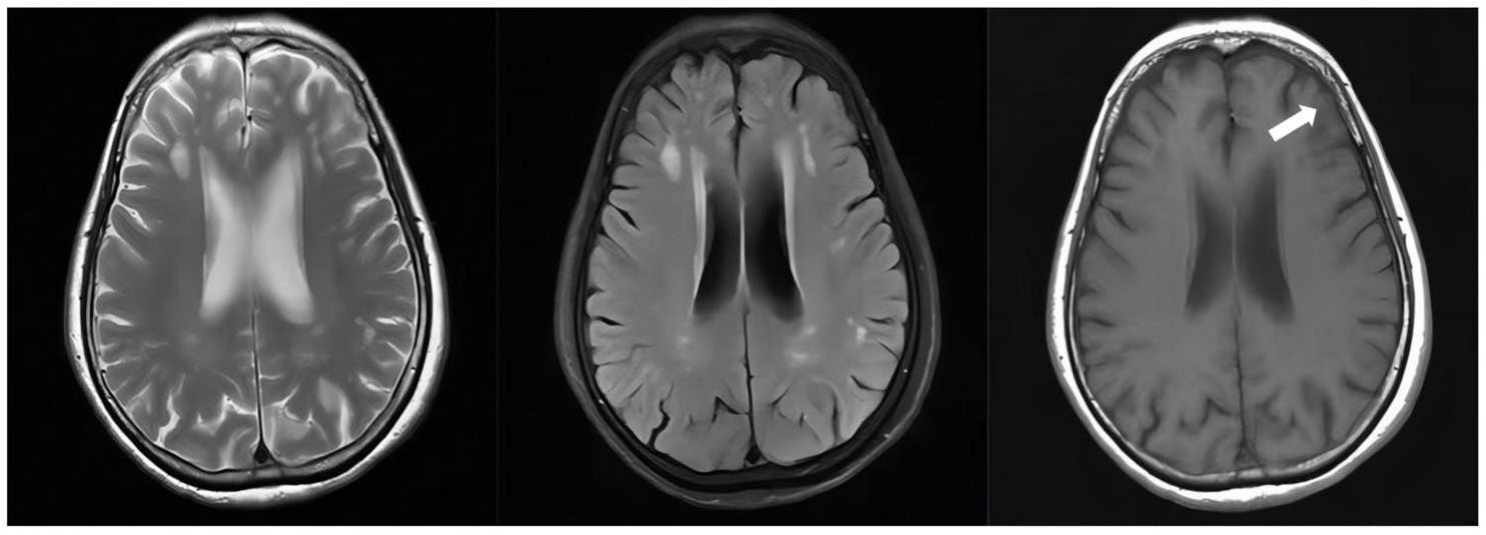
Follow-up unenhanced brain MRI scan on February 10, 2023(D + 371): The abnormally enhancing lesions in the right ambient cistern, left sylvian cistern, and adjacent brain parenchyma have improved.

After hospitalization for 185 days, the patient was discharged on August 8, 2022, with continued anti-TB therapy: isoniazid (0.6 g, qd, po), levofloxacin (0.6 g, qd, po), contezolid (0.4 g, bid, po), and vitamin B6 (50 mg, tid, po). The patient was reexamined regularly at follow-ups in the outpatient department. The following information was obtained at the follow-up visit on February 8, 2023 (D + 369): Routine blood test WBC: 3.89 × 10^9^/L; RBC: 3.86 × 10^12^/L, Hb: 112 g/L, PLT: 163 × 10^9^/L; Biology chemistry: ALT: 11.5 U/L, AST: 17.0 U/L, TBIL: 7.10umol/L, DBIL: 0.70umol/L, K: 3.9 mmol/L, TP: 53.9 g/L, ALB: 39.6 g/L, BUN: 3.96 mmol/L, CR: 62.0umol/L, UA: 262umol/L; Cerebrospinal fluid: intracranial pressure 140mmH2O; Profile: clear and colorless; CL: 125 mmol/L, ADA: 0.1 U/L, Glu: 2.74 mmol/L, Pr: 0.29 g/L, WBC 4 × 10^6^/L; Pandy test: negative; Tryptophan test: negative; The patient generally improved and her weight increased by about 10 kg. Her diet, sleep, urine, and defecation were regular. Apart from an intermittent numbness of both lower extremities, which became milder than before, the patient had no other complaints of adverse symptoms. The patient’s initial anti-TB treatment course was 12 months, of which she received contezolid for about eight months. The relevant follow-up indices were stable, and we continue to recommend that she have regular follow-up visits after stopping the anti-TB drugs. The contrast-enhanced MRI of the brain showed continued improvement (Figures 4, 5).

## Discussion

Linezolid, an oxazolidinone drug, has been widely used in anti-TB therapy of rifampicin-resistant TB (RR-TB), multidrug-resistant TB (MDR-TB), extensively drug-resistant TB (XDR-TB), and drug-resistant, cases of severe and refractory TBM (2–4). Our case presented with severe and refractory TBM. Due to liver dysfunction, the patient was intolerant to rifampicin and pyrazinamide therapy; therefore, linezolid was administrated. Despite significant benefits, linezolid may be accompanied by various adverse effects. A meta-analysis reported the adverse effects of linezolid in the treatment of extensively drug-resistant tuberculosis: the pooled estimates for the rates of myelosuppression, peripheral neuropathy, optic neuritis, and gastrointestinal adverse events were 42.5%, 26.0%, 19.0%, and 35.0%, respectively (5). According to the *Consensus on linezolid in the treatment of tuberculosis (2022 update)* (2), hematologic parameters should be monitored weekly for one month after starting linezolid and repeated every two weeks. If anemia and thrombocytopenia progress, the drugs should be reduced or discontinued, and hematologic parameters should be closely monitored. After two months of treatment with linezolid, our patient also suffered the adverse events in line with the description in the above-mentioned literatures, such as peripheral neuropathy as well as myelosuppression. Linezolid was discontinued due to deteriorating anemia and failure of transfusion therapy. Subsequently, the red and white blood cell counts gradually improved, respectively. Based on this change, we deduced that the myelosuppression caused by linezolid which is usually reversible (Table S1 in Supplementary material). However, peripheral neuropathy is generally irreversible in most patients, even after long-term withdrawal of linezolid (7, 8). The peripheral neuropathy of our patient slowly improved after the discontinuation of linezolid. Considering that patients with TB require long-term treatment, adverse effects of linezolid are a big concern in our clinical practice and limit the use of linezolid.

Contezolid is a novel oxazolidinone drug designed to address the myelosuppression and MAO inhibition associated with linezolid. It can inhibit the formation of a functional 70S initiation complex required for bacterial replication and has a broad antibacterial spectrum against *Staphylococcus*, *Streptococcus*, *Enterococcus*, *Mycobacterium tuberculosis*, *nontuberculous mycobacteria*, and *Nocardia* (6, 9–12). A number of studies (13–15) have confirmed the safety and tolerability of contezolid in healthy Chinese subjects, and a phase III clinical trial (16) in China showed that the incidence of hematological adverse events induced by contezolid during 7–14 days of treatment was significantly lower than that induced by linezolid. In animal experiments, contezolid can effectively eliminate *Mycobacterium tuberculosis* in the lungs of mice (17). A recent study (18) compared the mitochondrial protein synthesis inhibition and activity of approved and novel oxazolidinones against clinically relevant mycobacterial pathogens. The mitochondrial protein synthesis (MPS) IC_50_/MIC_50_ and IC_50_/MIC_90_ ratios or selectivity index (SI) were calculated for all oxazolidinones as indicators of *in vivo* safety. Contezolid showed the highest SI among the approved oxazolidinones (contezolid SI = 27, linezolid SI = 16, and tedizolid SI = 2), suggesting contezolid as a potential candidate against tuberculosis.

After stopping linezolid, the adverse effects of our patient were alleviated, but CSF indicators were worsened; therefore, contezolid was used compassionately with the patient’s consent, and the improved CSF indicators were observed later (Table S1 in Supplementary material); CSF Protein also decreased from + D132 1.12 g/L↑ to + D153 0.79 g/L↑(Table S2 in Supplementary material). In the course of treatment, the patient had no obvious symptoms of mental disorders before the addition of cycloserine, and the symptoms of mental disorders gradually worsened after the addition of cycloserine, and the cycloserine was eventually discontinued, and the mental symptoms of the patient gradually improved after the discontinuation of the drug, and the use of ethambutol was eventually discontinued due to the patient’s complaint of gradual decrease in visual acuity during the course of antituberculosis treatment. With cycloserine and ethambutol discontinued due to mental symptoms, the combination regimen of isoniazid, levofloxacin, and contezolid demonstrated good anti-TB clinical efficacy and safety in this patient: the brain contrast-enhanced MRI showed continued improvement and no other adverse symptoms reported except for an intermittent numbness of both lower extremities. The successful treatment of our patient suggests that contezolid may have high blood–brain barrier permeability and possesses similar efficacy to linezolid but with significantly fewer adverse effects related to myelosuppression and peripheral neuropathy. Thus, contezolid may be a potential option to replace linezolid in future anti-TB treatment. In addition, our case is the first clinical evidence to demonstrate the efficacy and safety of long-term use of contezolid. This case has been on contezolid for eight months, and the longest reported case of contezolid use in the available published literature is a 101-year-old male patient with nosocomial vancomsycin-resistant *Enterococcus faecium* pneumonia who received contezolid for nearly four consecutive months, resulting in the disappearance of lesions, which may also suggest that contezolid has a good efficacy and safety profile in relevant infectious diseases (19). Our case may be helpful to provide a reference for further studies in the field of anti-TB treatment.

Currently, contezolid is only approved by the National Medical Products Administration for the treatment of complicated skin and soft tissue infections caused by *Staphylococcus aureus* (methicillin-sensitive and -resistant strains), *Streptococcus pyogenes,* or *Streptococcus agalactiae*. Its efficacy and safety in the treatment of TB, such as pulmonary TB and TBM, still need to be confirmed in several clinical trials. Whether linezolid can be replaced by contezolid in clinical practice to ensure better therapeutic efficacy and treatment adherence remains to be determined. After reviewing the national and international literatures, we did not find any published report on the use of contezolid in the treatment of TBM. In our patient, contezolid exhibited significant efficacy and better safety than linezolid in treating multidrug-resistant TBM. Our findings may be potentially significant, worthy of popularization, and imply a more extensive clinical application of contezolid in the future, and further tissue penetration-related pharmacokinetic studies, and randomised controlled clinical studies are needed.

## Data availability statement

The original contributions presented in the study are included in the article/Supplementary material, further inquiries can be directed to the corresponding author.

## Ethics statement

Ethical approval was not required for the studies involving humans because Retrospective case reports are not required to pass ethics committee scrutiny in China. The studies were conducted in accordance with the local legislation and institutional requirements. The participants provided their written informed consent to participate in this study.

## Author contributions

LW, ZX, and JZ are key members of writing the manuscript. TG and GW plotted Figures 1–4. CJ and LL searched academic papers. All authors contributed to the article and approved the submitted version.
