# Supplementary material for: Case report: Successful treatment with contezolid in a patient with tuberculous meningitis who was intolerant to linezolid
Source: Front Med (Lausanne). 2023 Oct 19;10:1224179. doi: 10.3389/fmed.2023.1224179 (PMC10621037; doi:10.3389/fmed.2023.1224179)
Supplement: Supplementary file 1 [file Table_1.docx]

***Supplementary Material***

**Case report: successful treatment with contezolid in a patient with tuberculous meningitis who was intolerant to linezolid**

**Zhe Xu^1†^, Jing Zhang^1†^, Tingting Guan^1^, Guichuan Wan^1^, Chao Jiang^1^, Linchuan Lang^1^, Lianzhi Wang^1,*^**

1. Harbin Chest Hospital, Heilongjiang Province, China

**^†^** These authors contributed equally to this work and share first authorship

*** Correspondence:** Lianzhi Wang, [1186433640@qq.com](mailto:1186433640@qq.com)

**Table S1.** Changes in hematologic parameters from D +86 (May 1, 2022)

| **Time** | **WBC×10^9^/L** | **RBC****×10^12^/L** | **HB g/L** | **PLT×10^9^/L** |
| --- | --- | --- | --- | --- |
| D +86 | 3.69↓ | 2.41↓ | 77↓ | 169 |
| D +94 ^a^ | 2.32↓ | 1.79↓ | 56↓ | 157 |
| D +95 | 3.05↓ | 2.25↓ | 70↓ | 128 |
| D +102 ^b^ | 2.38↓ | 1.86↓ | 55↓ | 113 |
| D +104 | 2.22↓ | 2.51↓ | 75↓ | 113 |
| D +111 | 2.61↓ | 2.57↓ | 74↓ | 200 |
| D +117 | 2.69↓ | 2.73↓ | 83↓ | 317 |
| D +132 ^c^ | 3.10↓ | 3.10↓ | 92↓ | 192 |
| D +146 | 2.63↓ | 3.19↓ | 94↓ | 168 |
| D +160 | 3.37↓ | 3.46↓ | 105↓ | 247 |
| D +236 | 4.48 | 3.29↓ | 105↓ | 175 |
| D +369 | 3.89↓ | 3.86 | 112 | 163 |

a: Two units of leukocyte-depleted red blood cell suspension were infused. B: Two units of leukocyte-depleted red blood cell suspension were infused again, and linezolid was stopped. C: Contezolid was added to the anti-TB therapy.

**Table** S**2.** Changes in routine CSF testing from D +90 (May 5, 2022)

| **Time** | **Cl**  **mmol/L** | **ADA**  **U/L** | **Glu**  **mmol/L** | **Pr**  **g/L** | **WBC**  **×10^6^/L** | **pressure**  **mmH₂O** |
| --- | --- | --- | --- | --- | --- | --- |
| D +90 | 117↓ | 2.9 | 1.75 ↓ | 0.72↑ | 40 ↑ | 70 ↓ |
| D +102 ^a^ | 120 | 2.6 | 1.87 ↓ | 0.65↑ | 25 ↑ | 80 |
| D +110 | 120 | 2.4 | 1.61 ↓ | 0.86↑ | 12 ↑ | 105 |
| D +125 | 122 | 2.4 | 1.40 ↓ | 1.08↑ | 36 ↑ | 85 |
| D +132 ^b^ | 121 | 2.6 | 1.38 ↓ | 1.12↑ | 25 ↑ | 82 |
| D +139 | 121 | 2.4 | 1.60 ↓ | 0.98↑ | 28 ↑ | 75↓ |
| D +153 | 120 | 2.2 | 1.94 ↓ | 0.79↑ | 8 | 70↓ |
| D +167 | 121 | 2.9 | 1.89 ↓ | 0.78↑ | 44 ↑ | 60↓ |
| D +181 | 121 | 2.4 | 1.83 ↓ | 0.68↑ | 30 ↑ | 60↓ |
| D +237 | 121 | 2.3 | 2.49 ↓ | 0.54↑ | 12 | 85 |
| D +369 | 125 | 0.1 | 2.74 | 0.29 | 4 | 140 |

a: Linezolid was stopped. B: Contezolid was added to the anti-TB therapy.
